# The Joint Link of the rs1051730 and rs1902341 Polymorphisms and Cigarette Smoking to Peripheral Artery Disease and Atherosclerotic Lesions of Different Arterial Beds

**DOI:** 10.3390/life13020496

**Published:** 2023-02-10

**Authors:** Sergey Zhabin, Victor Lazarenko, Iuliia Azarova, Elena Klyosova, Marina Bykanova, Svetlana Chernousova, Daniil Bashkatov, Ekaterina Gneeva, Anna Polonikova, Mikhail Churnosov, Maria Solodilova, Alexey Polonikov

**Affiliations:** 1 Department of Surgical Diseases №1, Kursk State Medical University, 3 Karl Marx Street, Kursk 305041, Russia; 2 Department of Surgical Diseases of Institute of Continuing Education, Kursk State Medical University, 3 Karl Marx Street, Kursk 305041, Russia; 3 Department of Biological Chemistry, Kursk State Medical University, 3 Karl Marx Street, Kursk 305041, Russia; 4 Laboratory of Biochemical Genetics and Metabolomics, Research Institute for Genetic and Molecular Epidemiology, Kursk State Medical University, 18 Yamskaya St., Kursk 305041, Russia; 5 Laboratory of Genomic Research, Research Institute for Genetic and Molecular Epidemiology, Kursk State Medical University, 18 Yamskaya St., Kursk 305041, Russia; 6 Department of Biology, Medical Genetics and Ecology, Kursk State Medical University, 3 Karl Marx Street, Kursk 305041, Russia; 7 Department of Medical Biological Disciplines, Belgorod State University, 85 Pobedy Street, Belgorod 308015, Russia; 8 Laboratory of Statistical Genetics and Bioinformatics, Research Institute for Genetic and Molecular Epidemiology, Kursk State Medical University, 18 Yamskaya St., Kursk 305041, Russia

**Keywords:** peripheral artery disease, genetics, single nucleotide polymorphism, genome-wide association study (GWAS), cigarette smoking

## Abstract

Genome-wide association studies (GWAS) have discovered numerous single nucleotide polymorphisms (SNP) contributing to peripheral artery disease (PAD), but their joint effects with risk factors like cigarette smoking (CS) on disease susceptibility have not been systematically investigated. The present study looked into whether CS mediates the effects of GWAS loci on the development of PAD and atherosclerotic lesions in different arterial beds. DNA samples from 1263 unrelated individuals of Slavic origin including 620 PAD patients and 643 healthy subjects were genotyped by the MassArray-4 system for rs1051730, rs10134584, rs1902341, rs10129758 which are known as PAD-associated GWAS loci. The rs1051730 polymorphism was strongly associated with an increased risk of PAD (*p* = 5.1 × 10^−6^), whereas rs1902341 did not show an association with disease risk. The rs1051730 polymorphism was associated with increased plasma levels of LDL cholesterol (*p* = 0.001), and conferred a greater risk of PAD in cigarette smokers than in nonsmokers (*p* < 0.01). Interestingly, the rs1902341T allele was associated with an increased risk of PAD in smokers and a decreased disease risk in nonsmokers. SNPs and CS were both linked to unilateral and/or bilateral atherosclerotic lesions of peripheral vessels, as well as the abdominal aorta, coronary, and cerebral arteries. The studied polymorphisms exert pleiotropic and cigarette smoking-mediated effects on atherosclerotic lesions of different arterial beds.

## 1. Introduction

Atherosclerotic lower-extremity peripheral artery disease (PAD) is a malignant cardiovascular disease responsible for high rates of morbidity, disability, and mortality in people worldwide [1]. PAD is defined by arterial stenosis or occlusion anywhere from the aortoiliac to the pedal arteries affected predominantly by the atherosclerotic process [2]. In recent years, increased attention on PAD has resulted in significant achievements in the understanding of pathological processes underlying disease development and complications such as dyslipidemia, inflammation, microvascular dysfunction disease, and thrombosis [1]. Although many risk factors are shared between PAD and other types of atherosclerosis, epidemiological data have increasingly demonstrated that peripheral artery disease deserves to be recognized as a distinct entity [1,3]. 

PAD is recognized as a multifactorial polygenic disorder caused by interactions between genetic and environmental factors [4,5]. This means that a better understanding of the molecular mechanisms underlying PAD can be achieved by jointly assessing the contributions of genetic and environmental factors to disease pathogenesis, thereby facilitating the introduction of an innovative approach to tailoring disease prevention and treatment, also known as personalized or precision medicine [6,7]. 

Several candidate gene, linkage, and genome-wide association studies (GWAS) as well as transcriptomics studies, have been undertaken and identified polymorphic genes and pathophysiological pathways contributing to the risk of PAD. GWAS have discovered a greater proportion of the genetic polymorphisms associated with PAD [5,8,9], which scan the entire genomes of large samples of patients and controls to identify which variants are overrepresented in diseased patients compared to unaffected subjects. As reported by the GWAS Catalogue (https://www.ebi.ac.uk/gwas/home, accessed on 23.05.2022), twenty genome-wide association studies have identified 238 single nucleotide polymorphisms significantly associated with susceptibility to peripheral artery disease in various populations of the world. However, non-replication of original findings discovered by GWAS and the inconsistence of their results have been common features of complex multifactorial diseases, including PAD [9,10,11]. Although genetic differences between populations may explain non-replication and inconsistent GWAS results, environmental risk factors and gene-environment interactions may account for a significant portion of such differences [12]. The joint effects of cigarette smoking, a major risk factor of PAD, and GWAS loci on disease susceptibility have not been systematically investigated. The aim of the present study was to investigate whether the effects of genetic variants, discovered by GWAS, on the risk of peripheral artery disease and atherosclerotic lesions of different arterial beds are triggered by cigarette smoking (CS), a key environmental risk factor for PAD.

## 2. Materials and Methods

### 2.1. Study Patients 

The study protocol conforms to the ethical guidelines of the 1975 Declaration of Helsinki. All participants provided written informed consent to participate in the study. The Regional Ethics Committee of Kursk State Medical University approved the study protocol (No 9, 10.12.2019). The study was conducted using DNA samples from 1263 unrelated individuals of Slavic origin (predominantly Russians), inhabitants of Central Russia. The study sample included two groups: the case group consisted of 620 patients with PAD and the control group of 643 subjects without chronic diseases. The control group was recruited in our previous genetic association studies of cardiometabolic diseases [13,14,15]. The control group included healthy individuals without any chronic disease. Patient recruitment and collection of DNA samples from PAD patients were conducted in the Vascular Surgery Division of Regional Clinical Hospital (Kursk) in the period between 2013 and 2019. The diagnosis of PAD was verified by experienced vascular surgeons as well as by using duplex scanning and angiography of the arteries of the lower extremities. All PAD patients were diagnosed with arterial stenosis due to the atherosclerotic process. All study subjects were interviewed regarding smoking (ever/never) status using a validated screener for environmental risk factors of age-related diseases [16]. Biochemical characteristics of plasma lipids were obtained from clinical records. Demographic, clinical, and biochemical characteristics of the study participants are summarized in Table 1. The mean age of PAD patients was higher than the mean age of healthy individuals (*p* = 0.001). The number of males was greater in the case group than in the control group (*p* < 0.0001). The case and control groups did not differ from each other with respect to body mass index and smoking status. Almost 30% of PAD patients suffered from coronary artery disease (CAD), and about 60% had hypertension.

### 2.2. Genetic Analysis 

Genomic DNA from study participants was isolated from whole blood samples containing 0.5M EDTA using standard phenol–chloroform extraction and ethanol precipitation. Genetic analysis was performed at the Research Institute for Genetic and Molecular Epidemiology, Kursk State Medical University. Single nucleotide polymorphisms associated with PAD in GWAS were randomly selected from the GWAS Catalog. Four SNPs such as rs1051730 (*CHRNA3*), rs10134584 (*GPR137C*), rs1902341 (*OSBPL10*), and rs10129758 (*TXNDC16*) were selected for this study. The MassArray-4 system (Agena Bioscience, San Diego, CA, USA) was used to genotype SNPs (primer sequences are available upon request). The quality of genotyping was evaluated in 95 randomly selected DNA samples that were not aware of the case or control status. Repeated genotyping of these samples using the same platform reached 100% concordance between initial and repeated results.

### 2.3. Statistical and Bioinformatics Analysis

The present study was conducted in accordance with STrengthening the REporting of Genetic Association Studies (STREGA) guidelines. Statistical power was estimated using the genetic association study (GAS) power calculator (https://csg.sph.umich.edu/abecasis/gas_power_calculator/, accessed on 14 March 2021). An association analysis (α = 0.05) could detect a genotype relative risk (GRR) of 1.30–1.50 with 88–99% power in the overall (620 cases and 643 controls) and a GRR of 1.40–1.7 with 80–99% power in the subgroup analyses. Fisher’s exact and chi-squared tests were used to assess the distribution of genotype frequencies according to the Hardy–Weinberg equilibrium (HWE) and the comparison of allele and genotype frequencies between study groups, respectively. The odds ratio (OR) and 95 percent confidence intervals (CI) of SNP-phenotype associations were calculated using multiple logistic regression with adjustments for cofactors. SNPstats statistical software [17] was used to assess the association of SNPs with the risk of PAD. Associations of genotype combinations (diplotypes) with the risk of PAD were evaluated by the chi-squared test and adjusted for multiple comparisons by the false discovery rate (FDR) method. The medians (Me) and interquartile ranges (Q1/Q3) were used to describe plasma lipids because they showed a deviation from the normal distribution as assessed by using the STATISTICA v13.0 software (TIBCO, USA). For a comprehensive functional annotation of the studied polymorphisms, online bioinformatics tools and resources such as GTEx Portal (https://www.gtexportal.org, accessed on 17 September 2022), eQTLGen database (https://www.eqtlgen.org, accessed on 20 September 2022), QTLbase (http://www.mulinlab.org/qtlbase/index.html, accessed on 03 October 2022), and VannoPortal (http://www.mulinlab.org/vportal, accessed on 13 December 2022) were used.

## 3. Results

### 3.1. Associations of Polymorphisms rs1051730 and rs1902341 with Peripheral Artery Disease

The genotype distributions for polymorphisms rs1051730 and rs1902341 were in Hardy–Weinberg equilibrium (*p* > 0.05). All study participants were homozygous wild-type carriers for SNPs rs10134584 and rs10129758, and minor allele frequencies were less than 1%, as has been compared with other European populations (https://www.ncbi.nlm.nih.gov/snp/, accessed on 04 December 2022). Hence, these polymorphisms were excluded from further analysis. Table 2 shows associations of polymorphisms rs1051730 and rs1902341 with PAD risk in our population. SNP rs1051730 was strongly associated with an increased risk of PAD (*p* = 5.1 × 10^−6^), whereas rs1902341 was not (*p* = 0.70). Allele rs1051730A and genotypes rs1051730G/A-A/A were found to be associated with PAD susceptibility regardless of sex, age, CAD, hypertension, and diabetes mellitus. Regression analysis showed no significant associations of the polymorphisms with the ankle-brachial index (ABI) in PAD patients (*p* > 0.05).

Association analysis stratified by smoking status revealed that rs1051730 confers the risk of PAD in both smokers and nonsmokers, but the magnitude of disease risk was higher in smoker patients than in nonsmokers (Table 2). Interestingly, allele rs1902341T and genotype rs1902341T/T showed association with increased risk of PAD in smokers but decreased risk or no risk in nonsmokers.

### 3.2. Join Effects of the Studied Polymorphisms on the Risk of Peripheral Artery Disease

We analyzed whether genotype combinations (diplotypes) are associated with PAD risk. As can be seen from Table 3, two diplotypes such as rs1051730G/G×rs1902341C/T and rs1051730A/A×rs1902341C/C were significantly associated with decreased and increased disease risk, respectively. In cigarette smokers, diplotypes rs1051730GA × rs1902341TT and rs1051730AA × rs1902341CT were found to be associated with increased risk of PAD in smokers, whereas diplotype rs1051730GG × rs1902341CC showed the protective effect against disease risk (Table 4). In the non-smoker group, two diplotypes such as rs1051730GA × rs1902341CC and rs1051730AA × rs1902341CC were associated with the risk of peripheral artery disease (Table 4).

### 3.3. Relationship between the Polymorphisms and Plasma Lipids

Patients with genotype rs1051730A/A had the increased levels of LDL cholesterol in plasma, as compared with the G/G and G/A genotypes (difference + 0.39 mmol/L 95% CI 0.16–0.63, *p* = 0.001, log-additive genotype model). This association remained statistically significant after adjustment for the cofactors. No significant associations of SNPs were found with total cholesterol, HDL cholesterol, and triglycerides.

### 3.4. The Impact of the Studied Polymorphisms on Other Atherosclerotic Lesions in PAD Patients

**Figure 1:**
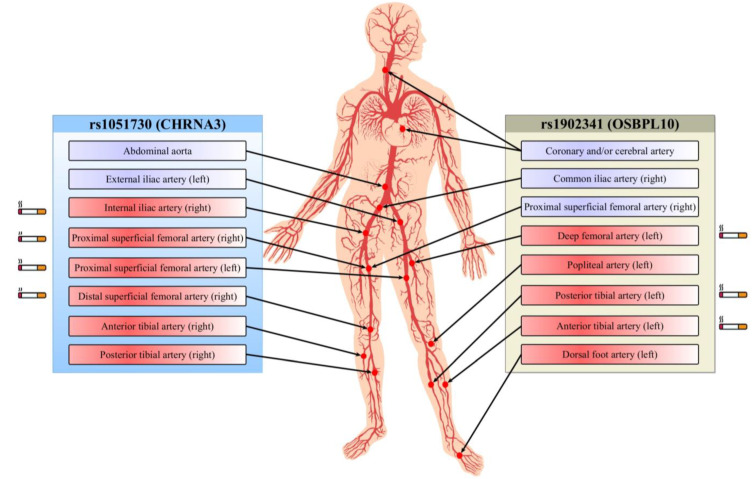
Relationship between polymorphisms and multiple arterial bed lesions. Full-colored blocks depict arterial stenosis identified by ultrasound scan (violet) and angiography (red). Smoking-mediated associations of SNPs with arterial stenosis are indicated by the cigarette icons.

The results of association analysis of the rs1051730 and rs1902341 polymorphisms with other atherosclerotic lesions in PAD patients are shown in Table 5 and summarized in Figure 1. SNP rs1902341 was associated with multifocal atherosclerosis in PAD patients (presence of coronary and/or cerebral artery atherosclerosis) regardless of patient’s sex and age (*p* = 0.019). SNP rs1051730 was associated with atherosclerosis of the abdominal part of the aorta (*p* = 7.0 × 10^−4^) and the stenosis of the left external iliac artery (*p* = 0.002), right posterior (*p* = 0.024), and anterior tibial arteries (*p* = 0.018). Notably, the rs1051730 polymorphism was associated with stenosis of some peripheral arteries in cigarette smokers, whereas no such association was found in nonsmokers. Smokers with the rs1051730 variant alleles had stenosis of proximal superficial femoral arteries on the right (*p* = 0.03) and left (*p* = 0.003) sides, internal iliac artery (*p* = 0.02), and right distal superficial femoral artery (*p* = 0.01). Furthermore, SNP rs1051730 was found to be associated with ultrasound-verified stenosis in the branch of the aortofemoral prosthesis on the right side (*p* = 0.03). The rs1902341 polymorphism showed association with stenosis of the right common iliac artery (*p* = 0.02), right proximal superficial femoral artery (*p* = 0.001), left popliteal artery (*p* = 0.02), and left dorsal foot artery (*p* = 0.01), regardless of smoking status. Meanwhile, SNP rs1902341 in cigarette smokers was associated with angiography-verified stenosis of deep femoral artery (*p* = 0.04), posterior (*p* = 0.03) and anterior (*p* = 0.02) tibial arteries on the left side.

### 3.5. Functional Annotation of the Studied Polymorphisms

In order to explore whether the studied polymorphisms impact the gene expression in the blood and arteries or represent eQTLs (expression quantitative trait loci) [18], the functional annotation was performed using several eQTL databases. Table 6 summarizes tissue-specific cis-eQTL (eQTLs located near the gene-of-origin) analysis for the polymorphisms. SNP rs1051730 is associated with expression of several genes in both tibial arteries (*CHRNA3, ADAMTS7*, and *PSMA4*) and whole blood (*PSMA4, IREB2, CTSH*, and *AGPHD1*). In particular, alternative allele rs1051730A is associated with decreased expression of *CHRNA3* (*p* = 3.1 × 10^−11^) and increased expression of *ADAMTS7* (*p* = 1.7 × 10^−7^) in the tibial artery. Furthermore, allele rs1051730A is associated with increased expression of *PSMA4* in the tibial artery (*p* = 0.0002), aorta (*p* = 0.000002), and whole blood (*p* < 0.0001). In the whole blood, the rs1051730A allele is associated with decreased levels of *IREB2* (*p* ≤ 0.0001) and *AGPHD1* (*p* = 4.6 × 10^−7^) as well as with increased levels of *CTSH* (*p* = 1.4 × 10^−26^), whereas the rs1902341 variant was associated with expression of *STT3B* (*p* = 0.0015), *OSBPL10-AS1* (*p* = 0.0027), *CMTM8* (*p* = 0.0089), and *SUGT1P2* (*p* = 0.0289). 

In addition, polymorphism rs1902341 was found to be associated with expression of *STT3B* in blood CD14+ monocytes (*p* = 0.0015), *OSBPL10-AS1* in dendritic cells (*p* = 0.003), *CMTM8* in blood CD8+ T cells (*p* = 0.009), and *SUGT1P2* in blood naive CD4+ T cells (*p* = 0.03), as can be seen from the QTLbase database. As can be seen from the VannoPortal, polymorphism rs1902341, despite being a synonymous SNP, is predicted as a likely pathogenic variant. In the aorta, polymorphism rs1902341 is associated with gene enhancer histone mark H3K4me1, related to weak transcription. SNP rs1051730 is also predicted as a likely pathogenic variant.

## 4. Discussion

The present study was the first to replicate that the rs1051730 and rs1902341 polymorphisms are PAD-associated loci, and the variants exert pleiotropic effects on atherosclerosis of different arterial beds. We also found that the impact of these polymorphisms on atherosclerotic lesions is modulated by cigarette smoking. In particular, a CS-dependent association between the rs1902341A allele and PAD was established for the first time: smokers have an increased disease risk, whereas nonsmokers have a decreased disease risk. The rs1902341 polymorphism was associated with atherosclerosis of multiple arteries, including peripheral ones such as the common iliac artery, proximal superficial and deep femoral arteries, popliteal artery, posterior and anterior tibial arteries (predominantly on the left side), as well as coronary and cerebral arteries. The rs1051730 variant was also associated with increased LDL cholesterol and stenosis of different arteries of the lower legs, iliac arteries, and abdominal aorta.

The eQTLGen Consortium’s large-scale genomics–transcriptomics data analysis revealed that the PAD-associated allele rs1051730A is strongly correlated with decreased expression of *IREB2* and *AGPHD1,* as well as increased expression of *PSMA4* and *CTSH* in whole blood (Table 6). Gene Ontology (GO) enrichment analysis shows that *IREB2* is an iron-responsive element-binding protein 2 involved in cellular iron ion homeostasis (GO:0006879) and the tricarboxylic acid metabolic process (GO:0072350). *AGPHD1* encodes hydroxylysine kinase, an enzyme catalyzing the GTP-dependent phosphorylation of 5-hydroxy-L-lysine [19]. *PSMA4* is a component of the 20S core proteasome complex, which is responsible for the ATP-dependent proteolytic degradation of ubiquitinated misfolded or damaged proteins [20], a part of the cellular stress response known as the unfolded protein response (UPR). Importantly, UPR activation plays an important role in the development and progression of atherosclerosis and occurs at all stages of the atherosclerotic process, from areas of intimal thickening, fatty streaks, and complex lesions [21,22]. Hence, it could be suggested that the rs1051730A allele, correlated with increased expression of PSMA4, may be linked to atherosclerosis through UPR activation.

CHRNA3 is an alpha-3 subunit of the nicotinic acetylcholine receptor (AChR), an integral membrane protein responding to the binding of acetylcholine that helps open an ion-conducting channel across the plasma membrane [23]. The α3 nAChR subunit is known to heteropentamerize with the β4 nAChR subunit and impact the responsiveness of cells to the actions of acetylcholine [23]. The polymorphism rs1051730 of *CHRNA3* showed a link to the PAD risk in our population, and the strength of this association is increased by CS, thereby confirming a smoking-related SNP-disease association originally discovered in GWAS performed by the GoLEAD and SUMMIT Consortiums [9]. Tobacco smoke has been shown to increase CHRNA3 expression at both the mRNA and protein levels in mice and humans [24,25], and this effect may be mediated by nicotine [26] or other chemicals such as benzo(a)pyrene [27]. On the one hand, cigarette smoking may mediate genetic risk for PAD attributed to the *CHRNA3* polymorphisms, as has been demonstrated in CAD [28]. On the other hand, the rs1051730-A allele is known to be strongly associated with smoking behavior traits such as nicotine dependence, heavy smoking, and cigarettes smoked per day [29,30]. Acetylcholine elicits endothelium-dependent vasodilatation [31]. In the tibial artery, the rs1051730-A allele is associated with decreased expression of *CHRNA3*, suggesting that the rs1051730-A allele may promote a decrease in AChR-mediated arterial dilatation by acetylcholine, thereby promoting vasospasm. Another mechanism by which CHRNA3 may be involved in the pathogenesis of PAD is the regulation of inflammation. AChR, being a part of the cholinergic anti-inflammatory pathway [32], plays a crucial role in mediating signals of the autonomic nervous system through decreasing the release of pro-inflammatory cytokines in neuronal and non-neuronal cells [33]. Pro-inflammatory effects of the polymorphism may be further demonstrated by the fact that allele rs1051730-A is also associated with increased expression of *ADAMTS7* (Table 4), a metalloproteinase involved in the modulation of vascular cell migration and matrix, processes ultimately leading to increased neointima formation and atherosclerosis [34].

The rs1902341 polymorphism has been found to be associated with the risk of PAD in Japanese population [35]. The SNP is located near the *OSBPL10* gene-encoding oxysterol-binding protein-related protein 10, which is thought to transport lipids and is involved in lipid counter-transport between the endoplasmic reticulum and plasma membrane [36]. OSBPL10 plays a role in the negative regulation of lipid biosynthesis [37], promotes apolipoprotein B-100 secretion by human hepatocytes, and binds cholesterol, 25-hydroxycholesterol, and acid phospholipids [38]. Apo-B100, a LDL receptor ligand linked to an increased risk of CAD [39], plays a direct role in the sub-endothelial retention of LDL [40]. The rs1902341 polymorphism is an interesting functional polymorphism with four eQTLs associated with *STT3B*, *OSBPL10-AS1*, *CMTM8*, and *SUGT1P2* expression, as well as being located in the area of epigenetic histone modification H3K4me1. Histone mark H3K4me1 (mono-methylation at the 4th lysine residue of the histone H3 protein) is thought to be associated with an active enhancer [41], providing an additional evidence for this variant’s functional significance. The mRNA levels of *OSBPL10-AS1* are negatively correlated with the presence of allele rs1902341T, which is associated with an increased risk of PAD in smokers and a decreased disease risk in nonsmokers. It could be proposed that the potential atherogenic effect of the rs1902341 polymorphism is attributed to the retention of LDL by the arterial wall as a result of increased hepatocyte secretion and blood circulation of apolipoprotein B lipoproteins. The later process is promoted by OSBPL10 [39], whose increased expression in carriers of the rs1902341T allele might be due to decreased levels of *OSBPL10*-*AS1* having the potential to hybridize mRNA *OSBPL10*, blocking its translation into the protein. 

One more potential mechanism for the association of the *OSBPL10* gene with atherogenesis may be related to its involvement in cholesterol metabolism. As mentioned above, OSBPL10 binds both cholesterol and 25-hydroxycholesterol. Oxysterols constantly exist in the human body as a result of cholesterol reactions with oxygen radicals or cholesterol hydroxylases in the cytochrome P450 family [42]. Numerous studies have shown that oxysterols derived from auto-oxidized cholesterol induce apoptosis or necrosis in vascular cells [43]. Oxysterols are formed in atherosclerosis foci due to LDL oxidation caused by the inflammatory response of cells to mechanical stress [44]. The oxysterol-to-cholesterol ratio in plaques is much higher than in normal tissues or plasma [43]. Experiments on mice have shown that high levels of 27-hydroxycholesterol contribute to the development of atherosclerosis by enhancing pro-inflammatory processes [45]. Furthermore, oxysterols, by causing defects in the packaging of phospholipids in the membranes of vascular endothelial cells, can increase cell permeability for LDL, resulting in atheroma formation [46]. As a part of UPR activation, STT3B mediates ubiquitin-dependent degradation of misfolded proteins in the endoplasmic reticulum via N-glycosylation of unfolded proteins [47]. *CMTM8*, whose expression is negatively correlated with allele rs1902341T, may also be linked with atherogenesis because chemokine-like factor 1 was found to play an essential role in the migration and proliferation of vascular smooth muscle cells, facilitating neointimal hyperplasia and atherosclerosis [48].

The study has some limitations. This study focused on a limited number of PAD-associated GWAS loci and their joint effects on atherosclerotic lesions only with a single environmental risk factor—cigarette smoking. We believe that by looking at a broader range of genetic variants and environmental factors, we will be able to paint a more complete picture of their relationship with the atherosclerosis in different arterial beds. Due to a lack of patient data on the number of cigarettes smoked (packs/year), it was not possible to assess the dose-dependent effects of cigarette smoking on the association between polymorphisms and disease susceptibility, as demonstrated in a study of Thorgeirsson with co-workers [49] for SNP rs1051730. In addition, associations between SNPs and atherosclerotic lesions are moderate or weak, and hence should be confirmed by independent studies. Since the studied SNPs do not encode amino acid changes, it is impossible to determine exactly which gene each polymorphism belongs to. The relationship between SNPs and expression levels of genes located in the same genomic region might be due to joint regulation by a common enhancer.

## 5. Conclusions

The present study was the first to replicate that the rs1051730 and rs1902341 polymorphisms are PAD-associated loci, and the variants exert pleiotropic effects on atherosclerosis of different arterial beds. In particular, the rs1902341 polymorphism was associated with atherosclerosis of multiple arteries, not just peripheral ones, while the rs1051730 variant was associated with increased LDL cholesterol and also with stenosis of different arteries of the lower legs, iliac arteries, and abdominal aorta. However, the studied polymorphisms exert pleiotropic and cigarette smoking-mediated effects on atherosclerotic lesions of different arterial beds, pointing out the importance of gene-smoking interactions in determining disease susceptibility. Experimental studies are required to elucidate the molecular mechanisms by which the studied polymorphisms influence both tissue-specific gene expression and the biological processes underlying the pathogenesis of atherosclerosis at different arterial beds.

## Figures and Tables

**Table 1:** Clinical and laboratory characteristics of study participants.

Characteristics		Healthy Controls (N = 643)	PAD Patients (N = 620)	*p*-Value
Age, mean ± standard deviation		60.66 ± 7.93	62.18 ± 9.13	**0.001**
Sex	Males, N (%)	391 (60.81)	532 (85.81)	**<0.0001**
	Females, N (%)	252 (39.19)	88 (14.19)	
Body mass index (kg/m^2^). mean ± standard deviation		27.07 ± 4.54	26.59 ± 6.36	0.12
Smokers *^a^*, N (%)		295 (47.2)	324 (52.3)	0.07
Coronary artery disease, N (%)		-	193 (30.63)	**-**
Hypertension, N (%)		-	369 (58.57)	**-**
TC (mmol/L), Me (Q1; Q3)		NA	5.07 (4.40; 5.60)	**-**
LDL-C (mmol/L), Me (Q1; Q3)		NA	3.20 (2.10; 4.50)	**-**
HDL-C (mmol/L), Me (Q1; Q3)		NA	1.18 (1.00; 1.57)	**-**
TG (mmol/L), Me (Q1; Q3)		NA	1.50 (1.11; 2.20)	**-**

PAD, peripheral artery disease; TC, total cholesterol; LDL-C, low-density lipoprotein cholesterol; HDL-C, high-density lipoprotein cholesterol; TG, triglycerides; *^a^* data on smoking status were not available from 18 subjects from the control group; statistically significant *p*-values are bolded. NA, not available.

**Table 2:** Associations of studied polymorphisms with the risk of peripheral artery disease in overall and cigarette smoking-stratified groups.

SNP ID(Gene)	Geno-Type, Allele	Genotype/Allele Frequencies. N (%)		*p*(*q*) ^*a*^	OR (95% CI) ^*b*^
		Healthy Controls (N = 643)	PAD Patients (N = 620)		
Entire groups					
rs1902341 (*OSBPL10*)	C/C	196 (30.5)	204 (32.9)	0.70 (0.70)	1.00
	C/T	342 (53.2)	302 (48.7)		0.91 (0.63–1.32)
	T/T	105 (16.3)	114 (18.4)		1.10 (0.68–1.77)
	T *^c^*	0.429	0.427	0.93 (0.93)	0.99 (0.85–1.16)
rs1051730 (*CHRNA3*)	G/G	309 (48.1)	228 (36.8)	**5.1 × 10^−6^** **(3.1 × 10^−5^)**	1.00
	G/A	285 (44.3)	302 (48.7)		**1.94 (1.36–2.75)**
	A/A	49 (7.6)	90 (14.5)		**2.75 (1.59–4.77)**
	A *^c^*	0.325	0.389	**0.0005** **(0.0015)**	**1.32 (1.13–1.55)**
Smokers					
rs1902341 (*OSBPL10*)	C/C	96 (32.5)	89 (27.5)	**0.05** (0.06)	1.00
	C/T	157 (53.2)	167 (51.5)		1.38 (0.83–2.28)
	T/T	42 (14.2)	68 (21.0)		**2.28 (1.16–4.48)**
	T *^c^*	0.408	0.468	**0.03** **(0.043)**	**1.27 (1.02–1.59)**
rs1051730 (*CHRNA3*)	G/G	141 (47.8)	114 (35.2)	**0.008** **(0.024)**	1.00
	G/A	134 (45.4)	165 (50.9)		**1.67 (1.04–2.66)**
	A/A	20 (6.8)	45 (13.9)		**2.97 (1.38–6.40)**
	A *^c^*	0.295	0.394	**0.0003** **(0.0015)**	**1.55 (1.22–1.97)**
Non-smokers					
rs1902341 (*OSBPL10*)	C/C	96 (29.1)	115 (38.9)	**0.05** (0.06)	1.00
	C/T	175 (53.0)	135 (45.6)		**0.52 (0.28–0.95)**
	T/T	59 (17.9)	46 (15.5)		0.45 (0.18–1.10)
	T *^c^*	0.444	0.383	**0.03** **(0.043)**	**0.78 (0.62–0.98)**
rs1051730 (*CHRNA3*)	G/G	155 (47.0)	114 (38.5)	**0.013** **(0.026)**	1.00
	G/A	149 (45.1)	137 (46.3)		**2.48 (1.30–4.72)**
	A/A	26 (7.9)	45 (15.2)		2.49 (0.87–7.07)
	A *^c^*	0.305	0.383	**0.003** **(0.006)**	**1.42 (1.12–1.80)**

*^a^* The *p*-values (*q*-values, false discovery rate) for associations of genotype/allele with the risk of PAD adjusted for sex, age, CAD, hypertension, and diabetes; *^b^* odds ratios and 95% confidence intervals (CI) for associations of a SNP with PAD risk; *^c^* relative frequency of a minor allele; statistically significant *p*- and *q*-values, ORs with 95% CIs are bolded.

**Table 3:** Association of genotype combinations (diplotypes) with the risk of peripheral artery disease.

№	Genotype Combination (Diplotype)	PAD Patients		Healthy Controls		OR (95% CI) ^*a*^	*p ^b^*	*q ^c^*
		N	%	N	%			
*G1*	rs1051730G/G × rs1902341C/C	74	11.9	104	16.2	0.70 (0.51–0.97)	**0.03**	0.07
*G2*	rs1051730G/G × rs1902341C/T	112	18.1	158	24.6	0.68 (0.52–0.89)	**0.005**	**0.02**
*G3*	rs1051730G/G × rs1902341T/T	42	6.8	47	7.3	0.92 (0.60–1.42)	0.71	0.77
*G4*	rs1051730G/A × rs1902341C/C	98	15.8	78	12.1	1.36 (0.99–1.87)	0.06	0.11
*G5*	rs1051730G/A × rs1902341C/T	149	24.0	159	24.7	0.96 (0.74–1.25)	0.77	0.77
*G6*	rs1051730G/A × rs1902341T/T	55	8.9	48	7.5	1.21 (0.81–1.81)	0.36	0.46
*G7*	rs1051730A/A × rs1902341C/C	32	5.2	14	2.2	2.45 (1.29–4.63)	**0.005**	**0.02**
*G8*	rs1051730A/A × rs1902341C/T	41	6.6	25	3.9	1.75 (1.05–2.92)	**0.03**	0.07
*G9*	rs1051730A/A × rs1902341T/T	17	2.7	10	1.6	1.78 (0.81–3.93)	0.14	0.21

*^a^* Odds ratio with 95% confidence intervals for particular genotype combination (crude analysis). *^b^* The *p*-values for association of particular genotype combination with PAD (Pearson’s chi-square test); *^c^ q*-values, adjusted *p*-values for multiple tests using Benjamini and Hochberg’s false discovery rate; Bold is statistically significant *p*- and *q*-values.

**Table 4:** Association of genotype combinations (diplotypes) with the risk of peripheral artery disease stratified by smoking status.

№	Genotype Combination (Diplotypes)	PAD Patients		Healthy Controls		OR (95% CI) *^a^*	*P ^b^*	*Q ^c^*
		N	%	N	%			
Smokers								
*G1*	rs1051730GG × rs1902341CC	28	8.6	47	15.9	0.50 (0.30–0.82)	**0.006**	**0.05**
*G2*	rs1051730GG × rs1902341CT	60	18.5	73	24.7	0.69 (0.47–1.02)	0.06	0.14
*G3*	rs1051730GG × rs1902341TT	26	8.0	21	7.1	1.14 (0.63–2.07)	0.67	0.86
*G4*	rs1051730GA × rs1902341CC	47	14.5	42	14.2	1.02 (0.65–1.60)	0.92	0.92
*G5*	rs1051730GA × rs1902341CT	82	25.3	73	24.7	1.03 (0.72–1.48)	0.87	0.92
*G6*	rs1051730GA × rs1902341TT	36	11.1	19	6.4	1.82 (1.02–3.24)	**0.04**	0.12
*G7*	rs1051730AA × rs1902341CC	14	4.3	7	2.4	1.80 (0.73–4.40)	0.27	0.49
*G8*	rs1051730AA × rs1902341CT	25	7.7	11	3.7	2.16 (1.04–4.47)	**0.03**	0.12
*G9*	rs1051730AA × rs1902341TT	6	1.9	2	0.7	2.40 (0.55–10.39)	0.35	0.53
Non-smokers								
*G1*	rs1051730GG × rs1902341CC	46	15.5	54	16.4	0.94 (0.61–1.44)	0.78	0.78
*G2*	rs1051730GG × rs1902341CT	52	17.6	78	23.6	0.69 (0.46–1.02)	0.06	0.18
*G3*	rs1051730GG × rs1902341TT	16	5.4	23	7.0	0.76 (0.39–1.47)	0.42	0.54
*G4*	rs1051730GA × rs1902341CC	51	17.2	36	10.9	1.70 (1.07–2.69)	**0.02**	0.09
*G5*	rs1051730GA × rs1902341CT	67	22.6	85	25.8	0.84 (0.58–1.22)	0.36	0.54
*G6*	rs1051730GA × rs1902341TT	19	6.4	28	8.5	0.74 (0.40–1.35)	0.33	0.54
*G7*	rs1051730AA × rs1902341CC	18	6.1	6	1.8	3.32 (1.34–8.93)	**0.01**	0.09
*G8*	rs1051730AA × rs1902341CT	16	5.4	12	3.6	1.51 (0.70–3.26)	0.29	0.54
*G9*	rs1051730AA × rs1902341TT	11	3.7	8	2.4	1.53 (0.62–3.76)	0.48	0.54

*^a^* Odds ratio with 95% confidence intervals for particular genotype combination (crude analysis). *^b^* The *p*-values for association of particular genotype combination with PAD (Pearson’s chi-square test); *^c^ q*-values, adjusted *p*-values for multiple tests using false discovery rate (FDR); bold is statistically significant *p*- and *q*-values.

**Table 5:** Relationship between studied polymorphisms and atherosclerotic lesions of different arterial beds verified by ultrasound scan and angiography.

Artery with Stenosis	rs1051730		rs1902341	
	Ultrasound	Angiography	Ultrasound	Angiography
Multifocal atherosclerosis (coronary and/or cerebral artery atherosclerosis)	1.89 (1.10–3.25) ^A^ *p* = 0.019	-	-	-
Aorta (abdominal part)	5.30 (1.85–15.19) ^A^ *p* = 7.0×10^−4^	-	-	-
Common iliac artery (right)	-	-	3.14 (1.05–9.35) ^D^ *p* = 0.02	-
External iliac artery (left)	7.19 (2.22–23.24) ^R^ *p* = 0.002	-	-	-
Proximal superficial femoral artery (right)	-	8.52 (1.25–57.83) ^R^ *p* = 0.03	5.41 (1.60–18.31) ^D^ *p* = 0.001	-
Internal iliac artery (right)	-	10.37 (1.39–77.61) ^D^ *p* = 0.02	-	-
Deep femoral artery (left)	-	-	-	4.73 (1.66–13.52) ^R^ *p* = 0.04
Proximal superficial femoral artery (left)	-	8.25 (2.16–31.46) ^R^*p* = 0.003	-	-
Distal superficial femoral artery (right)	-	9.82 (1.42–67.81) ^R^ *p* = 0.01	-	-
Popliteal artery (left)	-	-	-	2.07 (1.09–3.95) ^A^ *p* = 0.02
Posterior tibial artery (right)	-	4.02 (1.06–15.28) ^OD^ *p* = 0.024	-	-
Posterior tibial artery (left)	-	-	-	5.95 (1.21–29.37) ^R^ *p* = 0.03
Anterior tibial artery (right)	-	5.81 (1.09–30.97) ^OD^ *p* = 0.018	-	-
Anterior tibial artery (left)	-	-	-	7.90 (1.84–33.98) ^R^ *p* = 0.02
Dorsal foot artery (left)		-	-	8.67 (1.03–72.86) ^A^ *p* = 0.01

Odds ratio with 95% confidence intervals, *p*-values. Red shading indicates synergistic effect of SNP and cigarette smoking on the risk of stenosis in particular artery. Superscript letters depict genotypic association models: ^R^, recessive; ^D^, dominant; ^OD^, overdomonant; ^A^, log-additive.

**Table 6:** Tissue-specific *cis*-eQTL data for the rs1902341 and rs1051730 polymorphisms.

SNP ID	Allele Assessed	Changes in Gene Expression	Whole Blood *^a^*		Whole Blood *^b^*		Tibial Artery *^b^*	
			*q*	*Z*	*p*	*NES*	*p*	*NES*
rs1902341	C	*STT3B*	0.0015 *	−0.0183 *	-	-	-	-
	T	*OSBPL10-AS1*	0.0027 *	−3.0015 *	-	-	-	-
	T	*CMTM8*	0.0089 *	−0.1641 *	-	-	-	-
	-	*SUGT1P2*	0.0289 *	0.2276 *	-	-	-	-
rs1051730	A	*CHRNA3*	^**-**^	-	-	-	**3.1 × 10^−11^**	−0.35
	A	*ADAMTS7*	^**-**^	-	-	-	**1.7 × 10^−7^**	0.17
	A	*PSMA4*	**1.2 × 10^−90^**	20.2	**1.5 × 10^−9^**	0.09	**0.0002**	0.08
	A	*IREB2*	**3.3 × 10^−310^**	−41.4	**0.0001**	−0.07	-	-
	A	*CTSH*	**1.4 × 10^−26^**	10.7	-	-	-	-
	A	*AGPHD1*	**4.6 × 10^−7^**	−5.0	-	-	-	-

*^a^* False discovery rate and Z-score statistics for SNP’s *cis*-eQTLs in whole blood obtained from the eQTLGen database; *^b^ p*-value and NES (normalized effect size) statistics for SNP’s eQTLs in whole blood and tibial artery obtained from the GTEx portal; * statistics for SNP’s eQTLs (type of blood cell is mentioned in the text) obtained from the QTLbase database: *p*-value and Z-score or *beta* estimate. Bold is statistically significant p- and q-values

## Data Availability

Data supporting reported results are available upon request.
